# Sleep Fosters Odor Recognition in Children with Attention Deficit Hyperactivity Disorder but Not in Typically Developing Children

**DOI:** 10.3390/brainsci12091182

**Published:** 2022-09-02

**Authors:** Manuel Munz, Christian Dirk Wiesner, Meike Vollersen-Krekiehn, Lioba Baving, Alexander Prehn-Kristensen

**Affiliations:** 1 Central Outpatient Department, Center for Integrative Psychiatry, School of Medicine, Christian Albrecht University Kiel, 24105 Kiel, Germany; 2 Department of Child and Adolescent Psychiatry and Psychotherapy, Center for Integrative Psychiatry, School of Medicine, Christian Albrecht University Kiel, 24105 Kiel, Germany; 3 Department of Clinical Psychology and Psychotherapy, Institute of Psychology, Christian Albrecht University Kiel, 24118 Kiel, Germany; 4 Department of Psychology, MSH Medical School Hamburg, University of Applied Sciences and Medical University, 20457 Hamburg, Germany

**Keywords:** odor memory, attention deficit hyperactivity disorder, typically developing children, sleep-associated memory consolidation, biomarker, odor perception, dopaminergic dysfunction, neurodevelopment, information processing, learning

## Abstract

Prior experience represents a prerequisite for memory consolidation across various memory systems. In the context of olfaction, sleep was found to enhance the consolidation of odors in adults but not in typically developing children (TDC), likely due to differences in pre-experience. Interestingly, unmedicated children with attention deficit hyperactivity disorder (ADHD), a neurodevelopmental condition related to dopamine dysfunction, showed lower perceptive thresholds for odors, potentially allowing for more odor experience compared to TDC. We investigated sleep-associated odor memory consolidation in ADHD. Twenty-eight children with ADHD and thirty age-matched TDC participated in an incidental odor recognition task. For the sleep groups (ADHD: *n* = 14, TDC: *n* = 15), the encoding of 10 target odorants took place in the evening, and the retention of odorants was tested with 10 target odorants and 10 distractor odorants the next morning. In the wake groups (ADHD: *n* = 14, TDC: *n* = 15), the time schedule was reversed. Odor memory consolidation was superior in the ADHD sleep group compared to the TDC sleep and the ADHD wake groups. Intensity and familiarity ratings during encoding were substantially higher in ADHD compared to TDC. Sleep-associated odor memory consolidation in ADHD is superior to TDC. Abundant pre-experience due to lower perceptive thresholds is suggested as a possible explanation. Olfaction might serve as a biomarker in ADHD.

## 1. Introduction

Sleep fosters the formation of long-term memory by integrating newly encoded information traces into pre-existing memory systems [1]. This is most accurately understood for declarative memory: verbal representations of newly acquired memory traces are temporarily stored in the hippocampus and most effectively integrated into related neocortical memory systems during post learning (slow wave) sleep [2]. Sleep has been consistently reported to foster the consolidation of declarative memory more than wakefulness [1]. This contrast between sleep and wake offline consolidation is even more pronounced during development, when levels of slow-wave sleep are transiently higher [3,4,5]. On the contrary, the beneficial effect of sleep on motor memory performance is less clear in typically developing children (TDC) [3,5], supposedly due to less pre-existent motor experience. Odor memory formation and particularly sleep-associated consolidation has been less extensively studied compared to declarative or motor memory. Associations between odors and episodic memories appear to be strong and long-lasting [6]. However, responses to odors are not inherent and depend on experience [7] or repetition [8]. In this context and in the same line with motor memory, odor memory consolidation was found to be supported by sleep in adults but not in children, and the familiarity of odors was positively correlated with post-sleep odor recognition [9].

With a prevalence of 5–7%, attention deficit hyperactivity disorder (ADHD) [10], characterized by developmentally inappropriate levels of inattention, hyperactivity, and impulsivity [11], ranges among the most common childhood neuropsychiatric conditions. In addition to genetic factors [12], the interplay between stress, anxiety, and immune dysregulation in ADHD, resulting in a disrupted neuroendocrine stress response in ADHD, has been discussed to be involved in disordered neurodevelopment recently [13]. Structural imaging data have suggested that a delay in cortical maturation, in particular in frontal regions, might underlie this neurodevelopmental disorder (NDD) [14,15]. Besides structural findings, functional neural network abnormalities, including most prominently fronto-striatal, fronto-parieto-temporal, fronto-cerebellar, and fronto-limbic networks, were found [16,17]. Since sleep oscillatory activity is believed to parallel cortical maturation [18,19], alterations in slow [20,21] and theta oscillatory activity, linked to cognitive deficits, such as attenuated inhibitory control, were also interpreted as a delay in brain maturation [22]. There is considerable heterogeneity in cognitive impairments, which may be underpinned by different pathophysiological pathways [23]. Clinical diagnosis, so far based on clinical observation, continues to challenge clinicians considering the variability of clinical presentations [24]. While there is also a considerable overlap of symptoms with other NDDs, such as autism spectrum disorders, the identification of specific and biological developmental alterations in ADHD could unlock the potential of novel diagnostic markers and new treatment targets, such as neuroprotective agents [25]. Stimulant medications are first line treatment of ADHD, reducing the severity of core symptoms in up to 70% of patients by influencing dopaminergic signaling [26]. While a number of cognitive deficits, including goal directed behaviors, so-called executive functions (“EF”), are also present in other NDDs, stimulant treatment specifically leads to the normalization of under-activation in the inferior prefrontal cortex and the insula during the most consistently replicated EF tasks, such as response inhibition in ADHD [27]. On a neurochemical level, dopamine metabolism is crucial for EF through its neuromodulatory influence on fronto-striatal and related networks. Dopaminergic dysfunction, which can be partly cured by stimulants, is involved in the underlying pathophysiology of ADHD [28]. 

Likewise, altered olfaction has been found in other dopamine-related neuropsychiatric conditions, such as Parkinson’s disease [29]. Consequently, olfactory processing, in which dopaminergic signaling is significant too, was studied in the context of ADHD recently. Consistently lower olfactory thresholds have been found in ADHD compared to TDC [30,31,32]. There were no more differences in olfactory thresholds between ADHD and TDC when the first-line medication in ADHD, methylphenidate, which modulates dopamine metabolism, was used [31,32]. Additionally, an increase in volume of the olfactory bulb was found in ADHD [31]. Striatal dopaminergic dysregulation was proposed to cause a decrease in the inner-bulb-mediated inhibition of neuronal signals and thus allow higher sensitivity to odors in ADHD [30,31]. 

Altered dopamine function is also thought to reduce sleep-related functionality in the prefrontal cortex (PFC) [33,34]. Slow oscillations (SO) originating from the PFC mediate the integration of newly encoded information into pre-existent memory systems [1,5]. These were found to be less functional in ADHD, paralleling reduced sleep-associated declarative memory consolidation [20,21]. This, in turn, can be compensated for by the external application of SO over the PFC [35,36] but not acoustic closed-loop stimulation [37] and there are hints that physical activity might interact with declarative memory consolidation too [38]. While these studies displaying deficits in ADHD cover the sleep-dependent consolidation of verbally represented information, there is some evidence available that sleep might support motor memory consolidation in ADHD as opposed to TDC [39]. 

While the initial sensory processing of odors appears to be characterized by lower thresholds for perception [30,31,32], the subsequent cascade of odor memory formation in ADHD and especially sleep-associated consolidation has not been investigated so far. Contrasting findings of inferior cognitive control [17] and sleep associated memory consolidation [20,21], lowered thresholds for odors might implicate superior memory consolidation within this sensory system because of the accumulation of more abundant pre-existent odor memories and could add further evidence to the system consolidation hypothesis [1] Additionally, superior sleep-associated memory performance in ADHD would argue that odor memory integration is independent of slow oscillations [1,20,21,35,36]. Declarative memory consolidation in TDC is superior compared to children with ADHD, but since TDC with higher olfactory thresholds performed worse in an incidental sleep-associated odor memory consolidation task compared to adults [9], which was also found for non-verbal motor memory consolidation [3,5], we hypothesized that sleep would foster sleep-related odor memory consolidation in children with ADHD as measured by recognition accuracy.

## 2. Materials and Methods

### 2.1. Participants

Twenty-eight boys diagnosed with ADHD according to DSM-IV-TR [40] were included in the study (mean age 10.5, range 9–12 years). Nine among them were diagnosed with the inattentive subtype, nineteen with the combined subtype, and sixteen participants also fulfilled the criteria of oppositional defiant disorder. Data of 30 typically developed children (TDC) at comparable age (mean age 10.6, range 8–12 years) were taken from another sample being published before elsewhere [9]. In that study, the exact same procedure was used. In order to confirm the diagnoses (ADHD group) or exclude any psychopathology (TDC), respectively, all children and their parents were interviewed using a German translation of the Revised Schedule for Affective Disorders and Schizophrenia for School-Age Children: Present and Lifetime Version (Kiddie SADS-PL) [41,42]. Moreover, the Child Behavior Checklist (CBCL) [43], a standardized questionnaire as part of the Achenbach System of Empirically Based Assessment (ASEBA) was filled out by parents to assess any psychiatric symptoms in their children. Controls were excluded if they displayed any mental issues. Exclusion criteria for all participants were: diagnoses other than ADHD or oppositional defiant disorder (ODD), any continuous medication other than stimulants for the ADHD group, or any medication in the controls, below the average intelligence quotient (IQ < 85), as measured by the Culture Fair Intelligence Test 20-Revised Version-CFT 20-R [44], or pubertal development >5 by means of the pubertal development scale (PDS) [45]. Participants on any regular medication other than stimulants were excluded. Children with ADHD on regular stimulants were instructed to refrain from medication at least 48 h prior to the experiment. Initially, 30 children with ADHD were recruited; however, two were excluded during the screening process. 

ADHD patients were randomly assigned to a sleep or wake group with 14 children in each group in same way as it was done before in case of TDC [9]. There were no differences in the estimation of IQ, age, pubertal stage in the ADHD and TDC sleep (15 children) and wake (15 children) groups. There was also no difference in age, pubertal stage, or IQ (*p* > 0.1). Consistent with our diagnostic interview, psychopathology by means of ASEBA-scores was highly different between ADHD and TDC. Sample characteristics are given in Table 1.

Written informed consent was obtained from all children and their caretakers and all children were reimbursed with a voucher for their participation. The study was approved by the ethics committee of the Medical Faculty of the University of Kiel and followed the Declaration of Helsinki. ADHD patients were recruited via the clinic and the outpatient unit of the Department of Child and Adolescent Psychiatry and Psychotherapy of the Center for Integrative Psychiatry in Kiel, Germany, or by newspaper advertisements through which typically developing children (TDC) were recruited as well. 

### 2.2. Experimental Design and Procedures

For the sleep groups, encoding of odors took place at 8 p.m., followed by a night of sleep, and recognition was tested during retrieval after a 12-h retention interval at 8 a.m. In the same way for the wake groups, odors were encoded at 8 a.m. and retested at 8 p.m. Wake group participants were told not to sleep during the 12-h interval. Participants were asked to refrain from eating or drinking flavored drinks at least 30 min prior to each testing session. Twenty odorants of the University of Pennsylvania Smell Identification Test (UPSIT, Sensonics, Inc., Haddon Heights, NJ, USA) [46] were used for the old-new memory recognition paradigm. Ten target odors (“old”) from four categories (fruit/food/non-food/spice/plant) and ten distractor odors (“new”) from the same categories were selected for the experiment. Encoding and retrieval sessions took place in an air-conditioned laboratory. In order to measure implicit memory, participants were not told that there would be a recall session after the wake or sleep interval. Instead, they were instructed that they would participate in a study investigating circadian effects on odor perception. On a 9-point analogue scale (ranging from 0 = “not at all” to 8 = “maximum”), children were asked to rate each of the ten target odors with respect to their intensity, pleasantness, unpleasantness, and familiarity during the implicit encoding session. No memory instructions were given and odors were solely described along the above-named rating scales (e.g., no odor names). In the same location with the same environmental conditions, target and distractor odors were presented in pseudorandomized order at retrieval sessions. In a surprise odor memory recognition task, participants were instructed to indicate whether they recognized an odor from the (secret) encoding session (“old”) or not (“new”), followed by intensity, valence, and familiarity ratings. Thereafter, participants were asked if they anticipated to take part in a memory study and those answering “yes” were asked with what degree of certainty they anticipated a memory task (from 0 = “not at all” to 100 = “absolutely sure”). Using a standard evening and morning protocol [47] at the end of the encoding or retrieval sessions, participants were asked to rate mood, tiredness, and arousal on a 6-point analogue scale (mood: 1 = “feeling down” to 6 = “feeling happy”, tiredness: 1 = “feeling tired” to 6 = “feeling refreshed”, arousal: 1 = “feeling tense” to 6 = “feeling relaxed”) in order to control for circadian influences. 

### 2.3. Statistical Analysis

Statistical analysis was performed with IBM SPSS Statistics, version 26, for Windows. Descriptive statistics are presented as mean and standard error of means: M(SE). Memory performance was calculated using the standardized recognition accuracy d’ (“d prime” = standardized hit rate minus standardized false alarm rate) [48]. Analysis of variance (ANOVA) was performed using a 2 × 2 factorial design with the between factors SLEEP (sleep vs. wake) and ADHD (adhd vs. tdc). For analyzing ratings of intensity, valence, familiarity, and confidence ratings during encoding the same 2 × 2 ANOVA design was applied. Ratings during retrieval were analyzed using a 2 × 2 × 2 repeated measure ANOVA with the between factors SLEEP and ADHD and the within factor TARGET (target vs. distractor). Mood, tiredness, and arousal ratings were analyzed using a 2 × 2 × 2 repeated measure ANOVA with the between factors SLEEP and ADHD and the within factor SESSION (encoding vs. retrieval). The subsequent comparisons of single means were performed by *t*-tests for dependent or independent samples. Effect sizes are presented as eta squared (*η*^2^) and Cohen´s *d*. To analyze whether odor perception could be used to discriminate between ADHD and TDC, we used binary logistic regression. The accuracy, sensitivity, and specificity of the classification were computed using the caret toolbox in R. Sample size calculation by G*Power, (Version 3.1.9.7, Düsseldorf, Germany) [49] based on the following parameters: *f* = 0.47 [9], α error = 0.05, *β* error = 0.8, Numerator df = 3, and number of groups = 4 revealed a required total sample size of 54.

## 3. Results

### 3.1. Memory Performance

**Figure 1:**
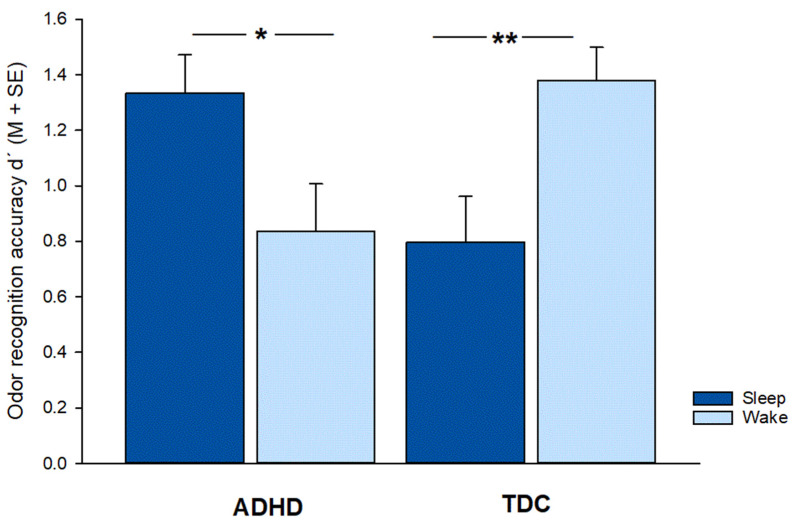
Odor recognition accuracy (d’) of the ADHD (attention deficit hyperactivity disorder; left) and TDC (typically developing children; right) sleep (dark blue) and wake groups (light blue); in ADHD odor recognition accuracy was higher in the sleep group compared to wake group, in TDC this pattern was reversed; M, mean; SE, standard error of means,*, *p* < 0.05; **, *p* < 0.01.

The analysis of recognition accuracy d’ revealed no main effect of ADHD (*F*(1,54) = 0, *p* = 0.999, *η*^2^ = 0) and no main effect of SLEEP (*F*(1,54) = 0.0, *p* = 0.952, *η*^2^ = 0). However, there was an ADHD x SLEEP interaction (*F*(1,54) = 14.0, *p* < 0.001, *η*^2^ = 0.206). An analysis of variance was decomposed with t-tests for independent samples, which revealed that the ADHD sleep group performed better than the ADHD wake group (sleep: 1.4(0.14); wake: 0.8(0.18); sleep vs. wake: *t*(26) = 2.5; *p* = 0.021, *d* = 1.04). As reported previously [9], TDC showed superior recognition in the wake group compared to the sleep group (sleep: 0.8(0.16); wake: 1.4(0.12); sleep vs. wake: *t*(28) = 2.8; *p* = 0.008, *d* = 1.04). With respect to performance in the sleep groups, recognition performance in the ADHD sleep group was better than the performance of the TDC sleep group (ADHD sleep: 1.4(0.14); TDC sleep: 0.8(0.16); ADHD vs. TDC: *t*(27) = 2.6; *p* = 0.015, *d* = 0.97). In contrast, considering the wake groups, performance in the TDC (wake) group was superior to the ADHD (wake) group (TDC wake: 1.4(0.12); ADHD wake: 0.8(0.18); TDC vs. ADHD: *t*(27) = 2.7; *p* = 0.012, *d* = 0.99). Results of memory recognition performance are given in Figure 1.

### 3.2. Odor Rating

#### 3.2.1. Encoding 

The highly significant main effects of ADHD revealed that children with ADHD rated odors as being more intense (ADHD: 6.55(0.18); TDC: 5.07(0.28); *F*(1,54) = 32.2, *p* < 0.001, *η*^2^ = 0.374) and more familiar (ADHD: 5.55(0.31); TDC: 4.38(0.23); *F*(1,54) = 9.6, *p* = 0.003, *η*^2^ = 0.149). There were no further significant effects of ADHD or SLEEP (*p* > 0.22, *η*^2^ < 0.028) or SLEEP x ADHD interactions (*p* > 0.24, *η*^2^ = 0.004) in the encoding session.

**Figure 2:**
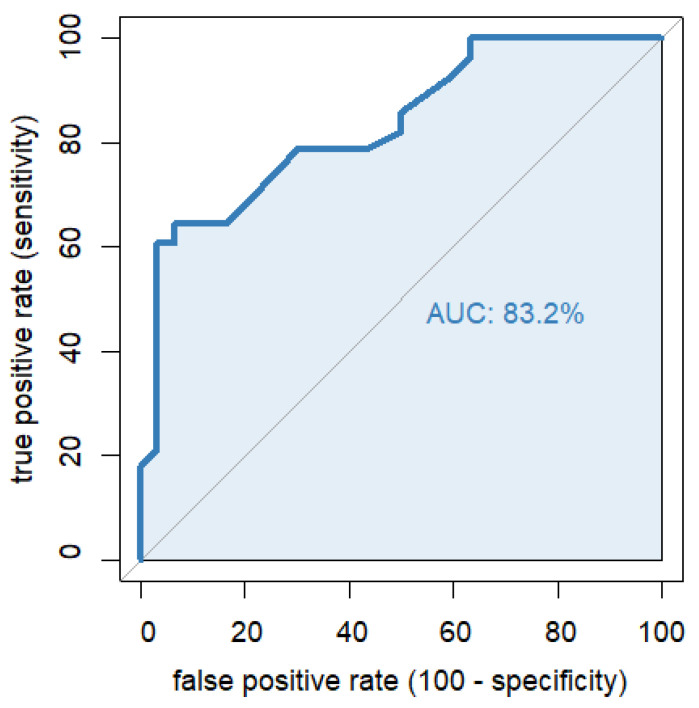
Receiver operating characteristic (ROC) curve for odor.

A binary logistic regression with the predictor “odor intensity” at the time of encoding and group membership as criterion showed highly significant results (χ^2^ = 22.5; df = 1; *p* < 0.001; Nagelkerke R2 = 0.429). Higher perceived intensity of the odors was predictive of ADHD (classification accuracy +/− 95% CI: 0.741 (0.610; 0.847), see also Table 2, Table 3 and Table 4). Children with ADHD and typically developing children can be very well distinguished on the basis of odor perception, which is also depicted in a receiver-operating curve in Figure 2. Sensitivity and specificity were even higher when intensity was combined with negative valence (for details see Supplement 1).

Intensity ratings during encoding as diagnostic predictor; the area under the ROC curve (AUC) refers to the overall diagnostic value; an AUC of 80–90% can be considered as excellent [50].

#### 3.2.2. Retrieval

During retrieval, as revealed by a main effect of ADHD, children with ADHD rated all odors as being more intense (ADHD: 6.65(0.18); TDC: 5.21(0.19); *F*(1,54) = 28.9, *p* < 0.001, *η*^2^ = 0.352), more unpleasant (ADHD: 3.38(0.31); TDC: 2.63(0.29); *F*(1,54) = 7.1, *p* = 0.01, *η*^2^ = 0.116) and displayed higher confidence in their ratings (ADHD: 6.28(0.21); TDC: 5.40(0.26); *F*(1,54) = 9.4, *p* = 0.003, *η*^2^ = 0.149). The main effects of TARGET showed that target odors were rated as more pleasant (targets: 4.82(0.19); distractors: 4.47(0.19); *F*(1,54) = 9.4, *p* = 0.003, *η*^2^ = 0.155), and more familiar (targets: 5.03(0.23); distractors: 4.27(0.22); *F*(1,54) = 34.6, *p* < 0.001, *η*^2^ = 0.390). The main effects of TARGET almost reached significance for confidence with higher ratings for targets (targets: 5.83(0.20); distractors: 5.62(0.19); *F*(1,54) = 3.07, *p* = 0.085, *η*^2^ = 0.054) and unpleasantness with higher ratings for distractors (targets: 2.67(0.18); distractors: 3.08(0.26); *F*(1,54) = 3.9, *p* = 0.052, *η*^2^ = 0.068). There were no further main effects of ADHD, SLEEP, or TARGET and no interactions (*p* > 0.15, *η*^2^ = 0.038). Means and standard errors of means of all ratings during encoding and retrieval divided into respective sleep and wake groups are displayed in Table 5.

### 3.3. Manipulation Check

Following the unexpected odor recognition task, participants were asked whether they suspected being part of a memory study. Among children with ADHD, five children of the ADHD sleep group and seven of the ADHD wake group as well as six children of the TDC sleep group and another six children from the TDC wake group had suspected memory recognition. Analysis with the Kruskal–Wallis test revealed that there was no difference in confidence ratings of the four groups among those who suspected being part of a memory study (*H*(3) = 5.601, *p* = 0.112). After excluding all participants who suspected being part of a memory study, odor recognition was recalculated. Again, the ANOVA revealed that there was no main effect of ADHD (*F*(1,30) = 0.3, *p* = 0.591, *η*^2^ = 0.010) and no main effect of SLEEP (*F*(1,30) = 0.1, *p* = 0.740, *η*^2^ = 0.004). However, the ADHD x SLEEP interaction remained significant (*F*(1,30) = 5.1, *p* = 0.031, *η*^2^ = 0.145). Post hoc independent sample t-tests revealed that the difference between the TDC sleep group and the ADHD sleep group (*t*(16) = 2.1; *p* = 0.055, *d* = 0.97) almost reached significance, while this was not true for the comparison between the ADHD wake and the TDC wake group (*t*(14) = 1.2; *p* = 0.265, *d* = 0.57). There was a statistical trend to worse performance of the TDC sleep group compared the TDC wake group (*t*(16) = 1.9, *p* = 0.071, *d* = 0.91), which was not true for the comparison of the performance of the ADHD sleep vs. ADHD wake group (*t*(14) = 1.3, *p* = 0.219, *d* = 0.63).

### 3.4. Control Variables

Ratings of mood, tiredness, and arousal served as a control for possible circadian effects. Repeated measure ANOVAS with the within factor SESSION encoding vs. retrieval) and the between factors ADHD (adhd vs. tdc) and CONDITION (sleep vs. wake) revealed the main effects of ADHD for each mood, tiredness, and arousal (*p* < 0.002, *η*^2^ = 0.157), because of higher ratings on each scale in ADHD children. However, there were no significant effects of SESSION (*p* > 0.058, *η*^2^ = 0.065) or CONDITION (*p* > 0.2, *η*^2^ = 0.028) for mood, tiredness, or arousal. For tiredness, there was a trend towards a SESSION x CONDITION interaction [*F*(1,54) = 3.6; *p* = 0.063, *η*^2^ = 0.063]. Mean differences between sleep and wake groups both for ADHD and TDC did not reach significance (*p* > 0.15). In order to exclude any influence of subjective ratings on memory performance, intensity, or familiarity ratings at encoding, which were largely different among children with and without ADHD, these variables were introduced as covariates in the ANOVA. ANCOVA of odor recognition likewise revealed no main effects of GROUP (*F*(1,48) = 0.2, *p* = 0.618 *η*^2^ = 0.005) or CONDITION (*F*(1,48) = 0, *p* = 0.870 *η*^2^ = 0.001) but again a highly significant ADHD X CONDITION interaction (*F*(1,48) = 15.2, *p* < 0.001 *η*^2^ = 0.240). Ratings for mood, tiredness, and arousal are given in Table 6.

## 4. Discussion

Using an incidental odor memory recognition paradigm, we found sleep-associated gains in odor memory consolidation in children with ADHD in contrast to age-matched TDC who showed attenuated memory consolidation during sleep. While sleep-associated consolidation in ADHD was investigated in the context of declarative and motor memory [20,21,35,38], this study is, to the best of our knowledge, the first to explore sleep-associated memory consolidation in this NDD. Our results both confirm findings of changes in initial odor perception in ADHD [30,31,32] and extend this body of research by providing an understanding of later stages of odor information processing.

### 4.1. Superior Consolidation of Odor Memory in ADHD Because of Abundant Pre-Experience?

Integrating newly acquired memory traces into pre-existing memory systems represents the core process of memory consolidation which is most effective during sleep in healthy populations and across various memory systems with abundant pre-existing memories through experience [1]. The functional deficits of slow oscillations in ADHD might explain reduced, sleep-associated declarative memory consolidation [20,21,35], whereas motor memory consolidation was better in children with ADHD compared to TDC, potentially due to abundant pre-experiences [39]. In the context of odor information processing, sleep was previously found to support memory consolidation in healthy adult individuals, and this finding of more effective odor memory consolidation compared to children has also been suggested to be linked to more abundant pre-experience [9]. In the present experiment, children with ADHD displayed a similar pattern of sleep-associated memory consolidation as adults did. In parallel, we replicated the findings from previous studies of lower thresholds by means of higher intensity ratings at encoding, putatively because of less effective dopaminergic inhibition in the olfactory bulbs [30,31,32]. Children with ADHD also rated odors as more familiar than TDC during encoding, both in the sleep (evening) and the wake groups (morning). We suggest that increased odor sensitivity might allow the build-up of pre-experience in children with ADHD as opposed to TDC, who have higher thresholds for olfaction. As indicated by higher familiarity ratings, accumulated pre-experience in turn might represent a favorable if not mandatory precondition for the process of memory consolidation, which is known to benefit from sleep [1]. This is, as mentioned above, most consistently described in the context of declarative memory and when subjects are told to memorize content after an interval filled with either sleep or wakefulness. In our rather small sample with small variance in familiarity ratings, we did not find correlations between familiarity ratings and subsequent consolidation, all of which would have further argued for improved consolidation on the basis of superior pre-experience.

Here, odors were presented, and participants were asked to rate various modalities, such as intensity, pleasantness, and familiarity, while no description in terms of verbal representations or associations of the odors was asked for. As a result, our incidental odor learning paradigm, with unannounced subsequent recognition following an interval filled with either sleep or wakefulness, allowed us to measure the recognition of pure olfactory sensation. When excluding participants who said that they believed to be taking part in a memory experiment (with diverging certainty ratings), the main results of superior sleep-associated memory consolidation of odors in ADHD remained unchanged. Additionally, serving as a control for circadian factors, subjective ratings of mood, tiredness, and arousal, which were quite different in ADHD and TDC but not different among the sleep or wake groups, had no influence whatsoever on odor memory consolidation. Hence, we can exclude that differences in memory consolidation can be ascribed to circadian factors.

We previously found less effective declarative memory consolidation linked to less functional slow oscillations [20,21,35]. As children with ADHD were found to outperform TDC in the context of olfaction, not only during initial but also during delayed information processing, the physiological pathways of superior sleep-associated odor memory consolidation would be interesting to explore. However, proposing that sleep fostered odor memory consolidation on the basis of superior pre-experience in ADHD, we cannot provide information about the contribution of various aspects of sleep (e.g., sleep stages or microevents such as sleep spindles) since we did not collect polysomnographic data. This should be considered for the design of subsequent studies aiming to understand the nature of sleep-associated odor memory processing in ADHD. Additionally, further functional imaging studies are warranted for the detailed elucidation of diverging underlying neural mechanisms among children with ADHD and TDC [32]. 

The majority of studies covering neuropsychological functions in ADHD report impairments or deficits, mainly in the context of executive functions, e.g., inhibition, vigilance, working memory, planning as well as in set shifting, selective attention, reaction time fluency, and decision making [24,33,34]. More specifically, deficits in sleep associated declarative memory consolidation have been found [20,21,35]. In contrast to various deficits, initial olfactory perception, e.g., chemosensory sensitivity and intensity [30,31,32], and sleep-associated memory consolidation as demonstrated in the current experiment appears to be superior in ADHD. On the one hand, these findings enable a more balanced and less deficit-focused picture of this neurodevelopmental condition (rather than ”disorder”), while, on the other hand, the clinical significance in terms of treatment remains elusive. 

### 4.2. Lowered Threshold for Olfaction a Useful Biomarker in ADHD? 

In contrast, improved olfaction in ADHD might become clinically significant in the context of diagnostics. Despite extensive research in the past decades, ADHD, based on the observation of behavioral symptoms, remains a major challenge, even for experienced clinicians [24]. For example, there is a remarkable variability of clinical presentations. Moreover, symptoms depend highly on context and cannot always be observed during clinical examination. Additionally, frequent and heterogeneous comorbidities will complicate the diagnosis even further. Despite extensive neurobiological research in the field of ADHD, its criteria defined by nosological systems: the current Diagnostic and Statistical Manual of Mental disorders (DSM, version 5) or the International classification of Diseases ICD-11 [11,51] become less precisely or specifically defined, emphasizing the need for the specialist’s experience or expertise. In this respect, our finding of highly significant differences in intensity and familiarity ratings of odors during initial encoding represents a relevant replication and extension of previous findings [30,31,32], suggesting olfaction as a simple, inexpensive, and, hence, promising biomarker in ADHD. Using only odor intensity as a predictor leads to a remarkable area under the curve (AUC) of 83.2 % in the ROC with sensitivity and specificity values comparable to the commonly employed oral glucose tolerance test for the diagnosis of gestational diabetes, for example [52]. Predictive accuracy (particularly specificity) was even higher when intensity was combined with unpleasantness (see Supplement 1: sensitivity = 0.786, specificity = 0.900; AUC = 88.2%) and might outperform clinical assessment as it is conducted in routine care [53,54]. Interestingly, increased sensitivity to odors seems to be quite specific in ADHD when compared to relevant differential diagnoses, such as autism, obsessive-compulsive disorder (OCD), and affective and anxiety disorders, in all of which olfaction appears to be attenuated or impaired [55]. The high specificity might pay out when regarding the potential of over-diagnosing ADHD [54]. Of course, the further development of olfaction as a biomarker in ADHD should entail standardized olfactometry and larger samples, including individuals with comorbidities or important differential diagnoses, such as autism-spectrum disorders, conduct disorder, OCD, and affective and anxiety disorders, and also consider potential developmental plasticity in olfaction. 

### 4.3. Strengths and Limitations

This study is the first to demonstrate superior sleep-associated odor memory in children with ADHD in contrast to TDC. Of course, the small sample size calls for larger confirmatory studies after our proof of principle. In this regard, one may assume our study as being not more than a pilot study. On the other hand, rigorous interviewing with strict inclusion criteria in children with ADHD according to research criteria and with no comorbidities except ODD and the exclusion of any disorder in the TDC group guaranteed homogeneous groups, reducing the probability of type 1 errors. A further strength is the use of a reliable, standardized test kit [46]. Due to our design measuring the consolidation of a pure odor memory trace apart from a verbal representation, initial encoding could not be measured. Our recognition measure is based on the assumption that encoding is not different among the sleep and wake groups. As an additional remark, the correlations of familiarity ratings at encoding with later memory performance did not reach significance, which would have further supported our hypothesis that prior experience enhances memory consolidation [9]. In further studies, stimulus material should be individually adjusted, so that subjective odor characteristics are comparable between all participants [31]. This would clarify whether in ADHD enhanced odor perception or altered higher-level odor processing is responsible for the findings. Finally, polysomnographic measures would have allowed a more fine-grained analysis of the sleep and memory interaction. Here, we would expect to find associations between odor memory improvements and sleep patterns that are linked to the consolidation of non-declarative or emotional memory representation (e.g., theta activity during REM sleep) [21,56]. A benefit from SO activity during SWS appears unlikely, since in all previous studies we did not find any evidence that in ADHD endogenous SO activity supported sleep-dependent memory consolidation [20,21,35].

### 4.4. Conclusions

In conclusion, sleep-associated odor memory consolidation is superior in children with ADHD compared to TDC. As indicated by higher familiarity ratings, more abundant pre-experience because of lower olfactory thresholds is suggested as a possible explanation. We suggest that attenuated dopaminergic inhibition in the olfactory bulbs [30,31,32] allows more odor memory traces to enter the hippocampal formation, where the actual redistribution to respective cortical regions takes place [1]. The sleep-associated consolidation of (verbally represented) visual or auditory stimuli, which are pre-processed in the entorhinal cortex [57], have been found to be attenuated in ADHD [20,21,35]. However, reduced inhibition in the olfactory bulbs might allow an abundance of odors to pass and enter the hippocampal formation via its efferences. In future studies, more detailed investigations including the measurement of initial encoding with individually adjusted stimulus material, neurophysiological data, including sleep spindles, connectivity analyses, and functional imaging with focus on hippocampus and associative cortices, are warranted to reveal the exact pathway and mechanisms of different odor memory consolidation and children with ADHD and TDC. Since odor processing and emotional learning share common neural pathways, it appears interesting to study whether in the context of fear conditioning, lowered perceptive thresholds can also be found. Increased sensitivity to fear-related stimuli would enable a more prompt and stronger pairing with noxious stimuli with respect to contextual fear conditioning, leading to “aberrant fear learning” as an integral component of many psychiatric conditions occurring as comorbidities of ADHD [58]. We also conclude that olfaction should be further developed as a promising biomarker in ADHD, being easy to use, inexpensive, and allowing quite reliable predictions of this neurodevelopmental condition.

## Figures and Tables

**Table 1:** Sample characteristics.

	ADHD					TDC					ADHD vs. TDC	
	Sleep Group *n* =14 M(SE)	Wake Group *n* =14 M(SE)	Sleep vs. Wake		Total *n* = 28 M(SE)	Sleep Group *n* = 15 M(SE)	Wake Group *n* = 15 M(SE)	Sleep vs. Wake		Total *n* = 30 M(SE)	*t*(56)	*p*
			*t*(26)	*p*				*t*(28)	*p*			
Age	10.5 (0.19)	10.4 (0.30)	0.4	0.704	10.5 (0.17)	10.7 (0.40)	10.5 (0.32)	0.4	0.696	10.6 (0.25)	0.4	0.720
PDS	3.4 (0.17)	3.1 (0.29)	1.1	0.295	3.3 (0.18)	3.3 (0.15)	3.1 (0.07)	1.2	0.242	3.2 (0.08)	0.4	0.652
IQ	101.9 (2.54)	106.7 (5.17)	0.9	0.390	104.1 (2,72)	108.4 (5.00)	111.2 (2.67)	0.5	0.599	110.1 (2.50)	1.6	0.111
CSHQ score	45.4 (1.15)	44.8 (1.70)	0.3	0.767	45.1 (1.01)	38.5 (0.70)	38.9 (0.72)	0.4	0.694	38.7 (0.50)	5.9	<0.001
ASEBA (T-values)												
Withdrawn/Depr.	62.1 (1.97)	62.4 (2.50)	0.5	0.912	62.3 (1.56)	53.5 (1.36)	52.1 (0.98)	0.8	0.410	52.8 (0.83)	5.5	<0.001
Somatic Complaints	59.9 (2.82)	59.2 (2.57)	0.4	0.868	59.5 (1.88)	53.1 (1.07)	52.2 (1.02)	0.6	0.533	52.7 (0.73)	3.5	<0.001
Anxious/Depressed	62.6 (1.62)	61.5 (2.70)	0.4	0.737	62.0 (1.55)	52.5 (1.10)	52.9 (1.13)	0.2	0.802	52.7 (0.78)	5.5	<0.001
Social Problems	62.1 (2.05)	63.1 (2.72)	0.4	0.771	62.6 (1.67)	52.2 (1.07)	52.3 (1.08)	0.0	0.956	52.2 (0.75)	5.8	<0.001
Thought Problems	57.6 (2.39)	56.4 (2.55)	0.4	0.731	57.0 (1.72)	51.2 (0.82)	50.6 (0.60)	0.6	0.559	50.9 (0.50)	3.5	<0.001
Attention Problems	66.9 (2.22)	68.3 (1.89)	0.3	0.629	67.6 (1.44)	50.8 (0.61)	50.9 (0.61)	0.1	0.939	50.9 (0.42)	11.4	<0.001
Rule-Breaking Beh.	64.2 (2.31)	61.9 (2.34)	0.2	0.494	63.1 (1.63)	51.3 (0.61)	50.4 (0.21)	1.3	0.188	50.8 (0.33)	7.6	<0.001
Aggressive Beh.	64.6 (2.45)	63.6 (2.53)	0.4	0.794	64.1 (1.73)	50.9 (0.50)	50.6 (0.45)	0.4	0.697	50.8 (0.33)	7.8	<0.001
Internal	62.8 (2.44)	62.3 (2.93)	0.4	0.897	62.5 (1.87)	48.4 (2.18)	47.1 (2.21)	0.4	0.686	47.8 (1.52)	6.1	<0.001
External	64.4 (2.79)	63.4 (2.52)	0.4	0.792	63.9 (1.85)	43.3 (1.82)	42.7 (1.70)	0.2	0.812	43.0 (1.23)	9.5	<0.001
Total	66.4 (2.46)	66.3 (2.31)	0.5	0.967	66.4 (1.66)	44.9 (2.23)	43.2 (1.94)	0.6	0.562	44.1 (1.46)	10.1	<0.001

Means (M) and standard error of mean (SE) of sample characteristics. ASEBA, Achenbach System of Empirically Based Assessment; PDS, pubertal development scale; CSHQ, Children’s sleep habits questionnaire.

**Table 2:** Binary logistic regression.

	B	SE	Wald	df	*p*
Intensity	1.309	0.340	14.810	1	<0.001
Constant	−7.637	1.999	14.598	1	<0.001

B, unstandardized regression coefficient; SE, standard error; Wald, Wald ratio; *p*, *p*-value of Wald statistic.

**Table 3:** Classification of ADHD and TDC based on odor perception.

		Predicted		
		TDC	ADHD	% Correct
observed	TDC	22	8	73.3
	ADHD	7	21	75.0
	Total			74.1

ADHD, attention deficit hyperactivity disorder; TDC, typically developing children; % correct refers to the percentage of correctly allocated participants to either the ADHD or the TDC group.

**Table 4:** Classification accuracy of ADHD and TDC based on odor perception.

Classification Measure	Value
accuracy [95% CI]	0.741 [0.610; 0.847]
no information rate	0.517
*p*-value of ACC > NIR	<0.001
sensitivity	0.750
specificity	0.733
positive prediction value	0.7241
negative prediction value	0.7586
balanced Accuracy	0.741

ADHD, attention deficit hyperactivity disorder; TDC, typically developing children.

**Table 5:** Odor rating data.

			ADHD			TDC			ADHD vs. TDC		
			Sleep Group	Wake Group	Total	Sleep Group	Wake Group	Total			
			M(SE)	M(SE)	M(SE)	M(SE)	M(SE)	M(SE)	*F*(56)	*p*	*η2*
Encoding	Targets	Intensity	6.59 (0.27)	6.51 (0.25)	6.55 (0.18)	4.88 (0.23)	5.27 (0.28)	5.07 (0.18)	32.2	<0.001	0.374
		Pleasantness	5.41 (0.39)	4.76 (0.38)	5.09 (0.28)	4.64 (0.34)	4.79 (0.28)	4.72 (0.22)	1.1	0.290	0.021
		Unpleasantness	2.42 (0.38)	2.52 (0.44)	2.47 (0.28)	2.15 (0.31)	1.91 (0.29)	2.03 (0.21)	1.5	0.220	0.028
		Familiarity	5.66 (0.42)	5.45 (0.46)	5.55 (0.31)	4.17 (0.32)	4.58 (0.34)	4.38 (0.23)	9.4	0.003	0.149
Retrieval	Targets	Intensity	6.79 (0.29)	6.48 (0.30)	6.63 (0.21)	4.98 (0.26)	5.49 (0.30)	5.24 (0.20)	23.3	<0.001	0.302
		Pleasantness	4.94 (0.41)	4.69 (0.47)	4.81 (0.31)	4.65 (0.37)	5.01 (0.25)	4.83 (0.22)	0	0.967	0
		Unpleasantness	2.99 (0.37)	3.03 (0.41)	3.01 (0.27)	2.51 (0.34)	2.17 (0.28)	2.34 (0.22)	3.7	0.060	0.064
		Familiarity	5.24 (0.58)	5.36 (0.39)	5.30 (0.34)	4.43 (0.47)	5.12 (0.35)	4.78 (0.29)	1.3	0.254	0.024
		Confidence	6.56 (0.29)	6.29 (0.30)	6.42 (0.21)	5.23 (0.36)	5.33 (0.51)	5.28 (0.31)	9.3	0.004	0.143
	Distractors	Intensity	6.88 (0.24)	6.46 (0.24)	6.67 (0.17)	5.03 (0.26)	5.37 (0.30)	5.20 (0.20)	31.3	<0.001	0.367
		Pleasantness	4.46 (0.40)	4.38 (0.42)	4.42 (0.28)	4.45 (0.42)	4.57 (0.32)	4.51 (0.26)	0.1	0.807	0.010
		Unpleasantness	4.01 (0.81)	3.59 (0.41)	3.75 (0.45)	2.41 (0.31)	2.50 (0.31)	2.45 (0.22)	6.9	0.011	0.113
		Familiarity	4.42 (0.54)	4.51 (0.41)	4.46 (0.33)	4.03 (0.39)	4.15 (0.45)	4.09 (0.29)	0.7	0.404	0.013
		Confidence	6.39 (0.29)	5.89 (0.31)	6.13 (0.22)	4.84 (0.39)	5.43 (0.41)	5.13 (0.28)	7.8	0.007	0.126

M = Means, SE = standard error of mean, ratings ranged from 0 = “not at all” to 8 = “maximum”; ADHD, attention deficit hyperactivity disorder; TDC, typically developing children.

**Table 6:** Ratings of mood, tiredness, and arousal.

		ADHD		TDC	
		Sleep Group (M(SE)	Wake Group M(SE)	Sleep Group M(SE)	Wake Group M(SE)
Encoding	Mood	5.5 (0.17)	5.5 (0.20)	4.0 (0.24)	4.5 (0.17)
	Tiredness	4.3 (0.34)	3.7 (0.44)	3.1 (0.25)	3.2 (0.39)
	Arousal	4.6 (0.40)	3.6 (0.44)	3.7 (0.32)	2.5 (0.22)
Retrieval	Mood	5.4 (0.17)	5.6 (0.20)	4.1 (0.24)	4.2 (0.22)
	Tiredness	3.8 (0.45)	4.4 (0.41)	2.8 (0.24)	3.5 (0.32)
	Arousal	5.0 (0.21)	4.7 (0.29)	2.5 (0.34)	3.7 (0.23)

M = Means, SE = standard error of mean of Mood, Tiredness, and Arousal, ranging from 1 = “lowest degree” to 6 = “highest degree”; ADHD, attention deficit hyperactivity disorder; TDC, typically developing children.

## Data Availability

Due to ethical, legal, or privacy issues, data should not be shared.

## Supplementary Materials

The following supporting information can be downloaded at: https://www.mdpi.com/article/10.3390/brainsci12091182/s1, Figure S1: Receiver Operating characteristic curve (ROC) for odor intensity and negative valence as predictors; Figure S2: Odor perception distinguishes ADHD from TDC—decision boundary from binary logistic regression; Table S1: Classification accuracy of TDC and ADHD based on odor perception with “intensity” only; Table S2: Binary logistic regression between ADHD and TDC based on odor intensity and negative valence; Table S3: Classification of TDC and ADHD based on odor intensity and negative valence; Table S4: Classification accuracy of TDC and ADHD based on odor intensity and negative valence.
