# Supplementary material for: Sleep Fosters Odor Recognition in Children with Attention Deficit Hyperactivity Disorder but Not in Typically Developing Children
Source: Brain Sci. 2022 Sep 2;12(9):1182. doi: 10.3390/brainsci12091182 (PMC9496889; doi:10.3390/brainsci12091182)

Supplement 1: Developing olfaction as a biomarker suggestive of ADHD

a) Predicting ADHD on the basis of odor intensity rating.

In the main body of the present manuscript, we present a binary logistic regression with the predictor "odor intensity" regarding encoding and group membership as a criterion with already remarkable classification accuracy (Table S1).

Table S1: Classification accuracy of TDC and ADHD based on odor perception with "intensity" only.

classification measure	value
accuracy [95% CI]	0.741[0.610; 0.847]
no information rate	0.517
p-value of ACC > NIR	< .001
Sensitivity	**0.750**
Specificity	**0.733**
positive prediction value	0.7241
negative prediction value	0.7586
balanced Accuracy	0.741

b) Predicting ADHD on the basis of **odor intensity and negative valence** rating.

Adding negative valence ratings as a second modality of olfaction increased the predictive accuracy of olfaction with respect to ADHD. A binary logistic regression with the predictors "intensity" and "negative valence" of the odor in encoding and group membership as a criterion showed highly significant results ($\chi 2= 30.33$; df = 2; $p < .001$; Nagelkerke $R2= .543$). A higher perceived intensity of the odors and higher ratings with respect to the negative valence of the odors were predictive of ADHD (classification accuracy +/- 95% CI: 0.845 (0.726; 0.927), the area under the curve (AUC) of the Receiver operating characteristic curve (ROC) was also raised to 88,2%). The results of binary regression and ROC calculations are presented in Tables S2, S3, and S4 and Figures S1 and S2.

Table S2: Binary logistic regression between ADHD and TDC based on odor intensity and negative valence.

	b	se	Wald	df	p
Intensity	1.766	0.476	13.793	1	< .001
Valence	0.878	0.354	6.163	1	.013
Constant	-12.127	3.253	13.900	1	< .001

Table S3: Classification of TDC and ADHD based on odor intensity and negative valence.

		predicted		
		TDC	ADHD	% correct
observed	TDC	27	3	90,0
	ADHD	6	22	78,6
				84,5

Table S4: Classification accuracy of TDC and ADHD based on odor intensity and negative valence.

classification measure	value
accuracy [95% CI]	0.845 [0.726; 0.927]
no information rate	0.517
p-value of ACC > NIR	< .001
sensitivity	**0.786**
specificity	**0.900**
positive prediction value	0.880
negative prediction value	0.818
balanced Accuracy	0.843

Figure S1: Receiver Operating characteristic curve (ROC) for odor intensity and negative valence as predictors.

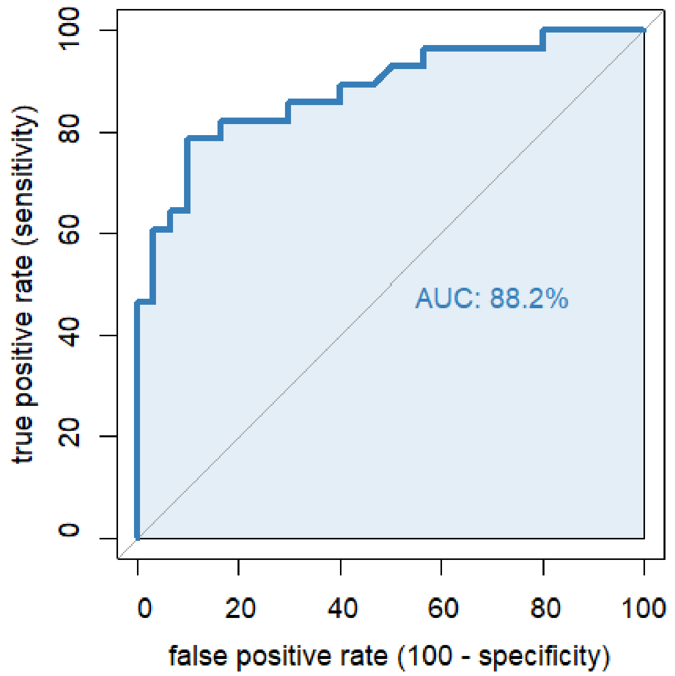

Figure S2: Odor perception distinguishes ADHD from TDC—decision boundary from binary logistic regression.

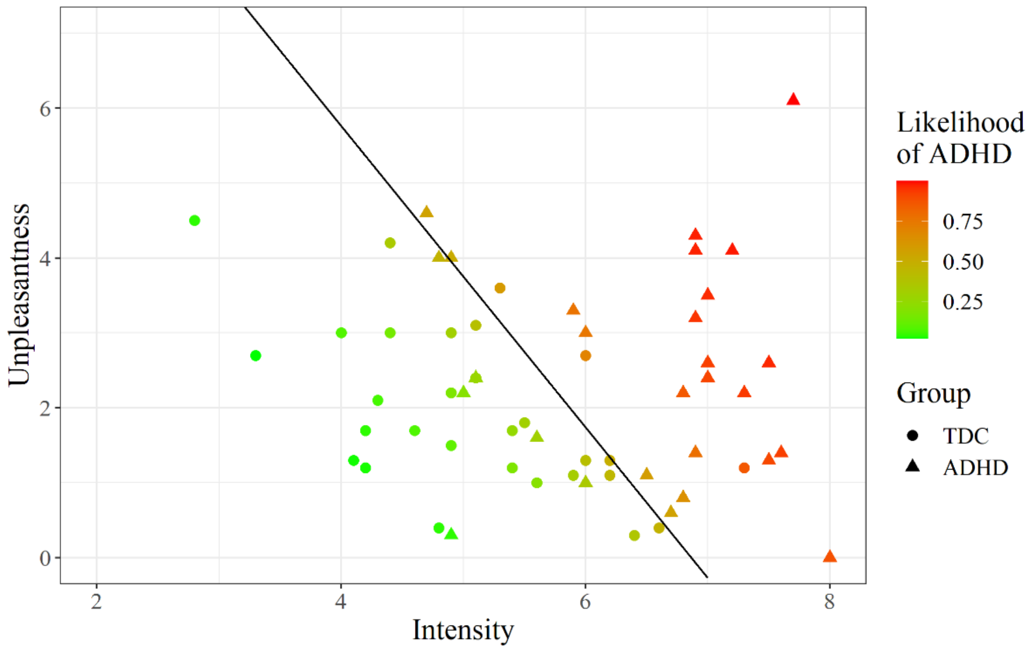

Supplement: Supplementary file 1 [file brainsci-12-01182-s001.zip › brainsci-1831125-supplementary.pdf]
